# A scoping review about social and emotional wellbeing programs and services targeting Aboriginal and Torres Strait Islander young people in Australia: understanding the principles guiding promising practice

**DOI:** 10.1186/s12889-020-09730-1

**Published:** 2020-10-29

**Authors:** Himanshu Gupta, Noemi Tari-Keresztes, Donna Stephens, James A. Smith, Emrhan Sultan, Sian Lloyd

**Affiliations:** 1 Wellbeing and Preventable Chronic Diseases Division, Menzies School of Health Research, Charles Darwin University, Darwin, NT Australia; 2 The Freemasons Centre for Male Health and Wellbeing – Northern Territory, Menzies School of Health Research, Charles Darwin University, Darwin, NT Australia; 3 National Drug Research Institute, Curtin University, PO Box 41096, Perth, NT 0811 Australia; 4 Menzies Centre for Health Policy, University of Sydney, PO Box 41096, Sydney, NSW 0811 Australia; 5 School of Public Health, Curtin University, PO Box 41096, Perth, NT 0811 Australia; 6 Orygen – Centre of Excellence for Youth Mental Health, 35 Poplar Road, Melbourne, VIC 3052 Australia

**Keywords:** Social and emotional wellbeing (SEWB), Aboriginal and Torres Strait islanders, Young people, Best/promising practices, Scoping review, Mental health

## Abstract

**Background:**

Multiple culturally-oriented programs, services, and frameworks have emerged in recent decades to support the social and emotional wellbeing (SEWB) of Aboriginal and Torres Strait Islander (Aboriginal) people in Australia. Although there are some common elements, principles, and methods, few attempts have been made to integrate them into a set of guidelines for policy and practice settings. This review aims to identify key practices adopted by programs and services that align with the principles of the *National Strategic Framework for Aboriginal and Torres Strait Islander Peoples’ Mental Health and Social and Emotional Wellbeing 2017–2023*.

**Methods:**

A comprehensive review of electronic databases and organisational websites was conducted to retrieve studies of relevance. Twenty-seven publications were included in the review. Next, we identified promising practices through a collaborative review process. We then used the principles articulated in the above-mentioned framework as the basis to complete a framework analysis. This enabled us to explore the alignment between current scholarship about SEWB programs and services with respect to the principles of the framework.

**Results:**

We found there was a strong alignment, with selected principles being effectively incorporated into most SEWB program and service delivery contexts. However, only one study incorporated all nine principles, using them as conceptual framework. Additionally, ‘capacity building’, ‘individual skill development’, and ‘development of maladaptive coping mechanisms’ were identified as common factors in SEWB program planning and delivery for Aboriginal people.

**Conclusion:**

We argue the selective application of nationally agreed principles in SEWB programs and services, alongside a paucity of scholarship relating to promising practices in young people-oriented SEWB programs and services, are two areas that need the urgent attention of commissioners and service providers tasked with funding, planning, and implementing SEWB programs and services for Aboriginal people. Embedding robust participatory action research and evaluation approaches into the design of such services and programs will help to build the necessary evidence-base to achieve improved SEWB health outcomes among Aboriginal people, particularly young people with severe and complex mental health needs.

## Background

Current literature provides a comprehensive and multi-faceted account about ways to address mental health concerns among Aboriginal and or Torres Strait Islander populations, with clear messages regarding preferred treatment options. One message is that effective mental health support must be embedded within a context of cultural understanding and knowledge [1, 2]. A key aspect of this cultural understanding is a framing of mental health within a broader conceptualisation of social and emotional wellbeing (SEWB).

For Aboriginal and Torres Strait Islander people SEWB includes specific and culturally defined relationships with family and community. These elements are embedded in roles and relationships within families, communities and spiritual connections to country, and ancestors [1, 3, 4]. Therefore, the structure of the sense of self for Aboriginal and Torres Strait Islander people is complex extending to family and clan group, within a complex set of relational bonds and reciprocal obligations. It may also incorporate ‘a profound sense of continuity through Aboriginal law and dreaming’ ([1]:56) .

Over the past few decades, multiple frameworks have emerged in relation to supporting the SEWB of Aboriginal and Torres Strait Islander people in Australia [1, 2, 5–7]. These visual frameworks are useful for understanding how these interconnected aspects of Aboriginal and Torres Strait Islander ways of being, knowing and doing can be incorporated within promising, and culturally informed, models of practice [1, 2, 8–14] and reflect the heterogenous nature of Aboriginal and Torres Strait Islander cultures in Australia. Table 1 outlines the key aspects incorporated into many of these frameworks.

**Table 1:** Summary table about the identified relevant frameworks

Reference	Name of the framework/model	Framework elements
[14] [10] [9] [7]	‘2004 SEWB framework’ National Strategic Framework for Aboriginal and Torres Strait Islander Peoples’ Mental Health and Social and Emotional Wellbeing 2017–2023; The National Strategic Framework for Aboriginal and Torres Strait Islander Peoples’ Mental Health and Social and Emotional Well Being 2004–2009)	**Nine guiding principles that underpin SEWB:** 1. Health as holistic 2. The right to self-determination 3. The need for cultural understanding 4. The impact of history in trauma and loss 5. Recognition of human rights 6. The impact of racism and stigma 7. Recognition of the centrality of kinship 8. Recognition of cultural diversity 9. Recognition of Aboriginal strengths
[1] [9]	‘Cultural Domains of Social and Emotional Wellbeing’	**Seven domains of SEWB:** 1. Connection to Body 2. Connection to Mind and Emotions 3. Connection to Family and Kinships 4. Connection to Community 5. Connection to Culture 6. Connection to Country 7. Connection to spirit, spirituality and ancestors
[11] [9]	‘Australian Government Implementation Plan 2007–2013’	**Key Result Areas:** 1. Social justice and across-government approaches 2. Population health approaches 3. Service access and appropriateness 4. Workforce 5. Quality improvement
[12] [9]	‘Revised national practice standards in mental health’	**Revised Practice Standards:** 1. Rights, responsibilities, safety and privacy 2. Working with people, families and carers in recovery-focused ways 3. Meeting diverse needs 4. Working with Aboriginal and Torres Strait Islander peoples, families and communities 5. Access 6. Individual planning 7. Treatment and support 8. Transitions in care 9. Integration and partnership 10. Quality improvement 11. Communication and information management 12. Health promotion and prevention 13. Ethical practice and professional development responsibilities
[13] [9]	‘Strong Spirit Strong Mind—Aboriginal Drug and Alcohol Framework for Western Australia 2011–2015′ (Drug and Alcohol Interagency Strategic Framework for Western Australia 2011–2015)	**Key Action Areas:** 1. Capacity Building 2. Working Together 3. Access to Services and Information 4. Workforce Development **Framework Key Strategic Areas:** 1. Focusing on prevention 2. Intervening before problems become entrenched 3. Effective law enforcement approaches 4. Effective treatment and support services 5. Strategic coordination and capacity building **The Seven Areas:** 1. Health 2. Family and Community Relationships 3. Aboriginal Law and Culture and Country 4. Land/Country 5. Grief and Loss 6. Livelihood/Money and Work 7. Legal
[8]	‘Quality Healing Program’	**Elements:** 1. Developed to address issues in the local community 2. Driven by local leadership 3. Have a developed evidence base and theory base 4. Combine Western methodologies and Indigenous healing 5. Understand the impact of colonisation and transgenerational trauma and grief 6. Build individual, family and community capacity 7. Proactive rather reactive 8. Incorporate strong evaluation frameworks
[2]	‘Representation of a culturally informed best practice pathway (pictorial)’	**Elements of the culturally informed best practice pathway:** **Wellbeing (being together):** 1. Culture, art, dance 2. Community, sport, work 3. Family, elders, friends 4. Services, housing, mental health, substance use
*(5)*	‘Wellbeing Framework’	**Core values:** 1. Wellbeing is supported by upholding peoples’ identities in connection to culture, spirituality, families, communities and Country 2. Wellbeing is supported by culturally safe primary healthcare services **Elements:** 1. Wellbeing is supported by locally defined, culturally safe primaryhealthcare services 2. Wellbeing is supported by an appropriately skilled and culturally competent healthcare team 3. Wellbeing is supported by holistic care throughout the lifespan 4. Wellbeing is supported by best practice care that addresses the particular needs of a community **Principles:** 1a: Creating culturally welcoming places 1b: Developing trusting relationships with clients and communities 1c: Understanding and accepting cultural diversity within communities 1d: Delivering flexible primary healthcare services both within and outside of healthcare facilities 2a: Ensuring that all staff are regarded by the community as culturally competent 2b: Equipping staff with suitable skills to support people with chronic disease 2c: Valuing and supporting Aboriginal and Torres Strait islander staff 2d: Developing effective cultural leadership 3a: Applying holistic approaches to address priorities determined with clients 3b: Life-course approach from pre-conception to post-mortality 3c: Ensuring appropriate resources are available to meet local priorities and needs 3d: Responding to family, community, cultural and spiritual responsibilities and obligations 4a: Utilising cultural and scientific evidence to provide best practice healthcare 4b: Ensuring that primary healthcare services are available, accessible and acceptable 4c: Empowering communities to be involved in determining local healthcare priorities 4d: Developing multi-disciplinary teams that support holistic care
*(6)*	‘Interrelated approach: SEWB, ACT, and strengths’	**SEWB:** 1. Spirituality 2. Respect **Strengths:** 1. Forgiveness 2. Integrity 3. Honesty 4. Courage 5. Empathy **ACT (Acceptance Commitment Therapy):** 1. Acceptance 2. Mindfulness
[6]	‘Interrelated approach: SEWB, ACT, and strengths’	**SEWB:** 3. Spirituality 4. Respect **Strengths:** 6. Forgiveness 7. Integrity 8. Honesty 9. Courage 10. Empathy **ACT (Acceptance Commitment Therapy):** 3. Acceptance 4. Mindfulness

One of the most comprehensive frameworks is the *National Strategic Framework for Aboriginal and Torres Strait Islander Peoples’ Mental Health and Social and Emotional Wellbeing 2017–2023* (henceforth SEWB Framework)*,* which has a foundation of development over many years (e.g., Ways Forward report, 2004 Framework) [10, 14]. It has nine guiding principles:

1. health as a holistic concept;
2. the right to self-determination;
3. the need for cultural understanding;
4. the impact of history in trauma and loss;
5. recognition of human rights;
6. the impact of racism and stigma;
7. recognition of the centrality of kinship;
8. recognition of individual and community cultural diversity; and
9. recognition of Aboriginal strengths [7, 9]

The culturally-oriented frameworks, programs, and services appear to have some common elements, principles and methods. Yet, few attempts have been made to distil these common elements into an integrated set of guidelines for policy and practice setting. There is also evidence that programmes introduced in an agreement with these principles are more likely to be successful than those not [15]. The mobility of some Aboriginal and Torres Strait Islander people, both within their language groups and into and out of remote, regional, and or urban areas, includes being connected as an ‘effective exercise of collective self-determination and cultural continuity’ ([4]:166). Promising practices recognise the need to address community collaboration and relationships and understand that ‘spirituality has been changed both through colonisation and purposeful connection to a range of other systems that sit alongside of, and together with, cultural and spiritual beliefs and values’ ([1]:60). Outcomes may be compromised if they do not address connections between mobility, family, culture and spirituality; although this alone does not measure success nor clearly determine links to measurable outcomes [16]. For example, The Centre of Best Practice in Aboriginal and Torres Strait Islander Suicide Prevention includes best practices for young people, families and communities on their website (https://www.cbpatsisp.com.au/).

Young people with severe or complex mental health issues experience a range of symptoms, behaviours, and triggers that are often extreme and life-threatening [17]. Dudgeon and Holland state that Aboriginal and Torres Strait Islander young people are five times more likely to die by suicide than their non-Aboriginal peers arising from a ‘complex web of interacting personal, social, political and historical circumstances’ ([4]:116). Stressors affecting individuals, families, and communities include interpersonal conflict; involvement with the justice system; poor access to education; and unemployment, all of which intersect with cultural and gender identity formation [18]. This can derive from complex disadvantage which is usually intergenerational and associated with the dispossession of land, policies of discrimination, and child removal associated with colonisation [19]. The social and cultural domains of Aboriginal and Torres Strait Islanders people’s health and wellbeing are often poorly reflected in the delivery of services [15]. This might impact and exacerbate barriers to help-seeking among young Aboriginal Australians, including shame, fear, and suspicion in accessing services [20]. This is further compromised by the need to navigate understandings of health, and health services and systems, between both Western and cultural paradigms [18].

Impact of social disadvantage due to lack of consistent access to quality food; malnutrition; alcohol and or drug use; interactions with legal systems; developmental or acquired cognitive impairment; and a range of chronic illnesses are often disproportionately noted among Aboriginal and Torres Strait Islander peoples, which frequently masks the early detection of mental health issues [21]. This requires collaborative and inter-sectoral solutions. However, Tsey et al. ([22]:36) suggest that social disadvantage does not automatically account for excess morbidity. They suggest that a deeper exploration of the social gradient in relation to patterns of morbidity and the amount of control people have over their lives - while ‘notoriously difficult to research, resource intensive and requiring a long-term commitment as well as the development of appropriate methodologies’ – has of great importance ([22]:37).

Thus, while we advocate for a collective approach to mental health for Aboriginal and Torres Strait Islander people, there is also a genuine need to focus on the individual with a specific focus on individual client pathways and effective individualised care. Armstrong et al. (2018), in recognising the higher prevalence of suicide rate among Aboriginal and Torres Strait Islander young people, state that it is more essential to make the individual feel comfortable, respected and cared for, than to do all the ‘right things’ and follow all the ‘rules’ when communicating with an Aboriginal and Torres Strait Islander person ([19]:5). For example, although often described as a useful tool, yarning, used without an understanding of how to ‘best engage and communicate mental health messages with Aboriginal adolescents’ ([20]:4) provides limited outcomes in addressing how Aboriginal adolescents conceptualise ‘their world, including mental health, family, relationships and identity [ibid21]’.

The strengths-based approach is a common principle in health research on Aboriginal and Torres Strait Islander people. It is a conceptual framework for approaching, developing, and intervening in their health and wellbeing. It also provides alternative ways of tackling health issues, especially mental health problems affecting this population [23].

In this study, we seek to identify key strategies adopted by programmes and services that adhere to the SEWB Framework, emphasising social and emotional well-being among young Aboriginal people and Torres Strait Islanders with a special emphasis on programmes and services addressing severe and complex mental health issues. The aim was to adopt a strengths-based approach [23] and consider the importance of implementing these principles in service design and to analyse them whether they align with the guiding principles.

It is important to note that Aboriginal and Torres Strait Islander people were represented on the review and authorship team in accordance with contemporary scholarship on Indigenous data sovereignty and to provide the opportunity for two-way learning and cultural capacity building. One team member contributed to the analytic and manuscript writing process, while the other team member undertook a critical review of the article. It should also be noted that none of the research team members were involved in any of the identified programs or services in this scoping review. The project was also discussed with members of the institutions’ Social and Emotional Wellbeing Aboriginal Advisory Committee, particularly with respect to cultural messaging and the adoption of a strengths-based approach.

## Method

A scoping review methodology was applied to map the relevant academic and grey literature to ensure a comprehensive coverage (breadth) of the available literature [24] in relation to the social and emotional wellbeing of Aboriginal and Torres Strait Islander young people and include a specific focus on severe and complex mental health needs. A scoping review is defined as a literature review which aims to map the key concepts underpinning a research area and the main sources and types of evidence available, and can be undertaken as a stand-alone project, especially where an area is complex or has not been comprehensively reviewed before [25].

**Fig. 1:**
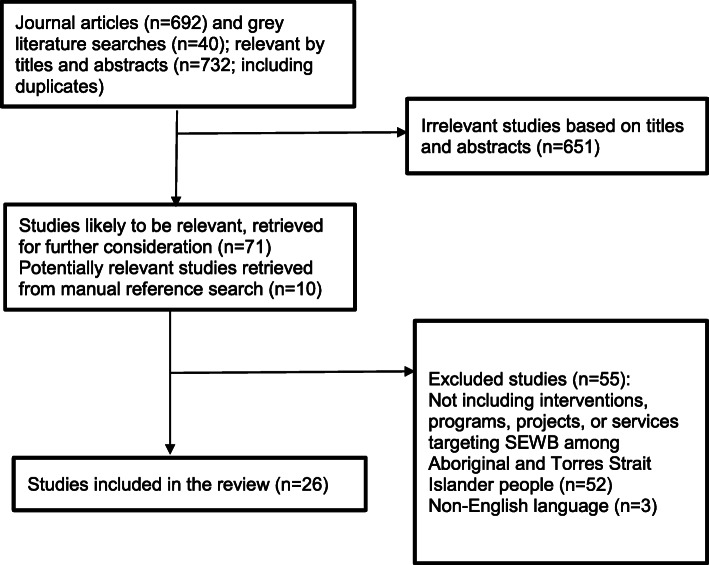
Results of search strategy

Building on a similar search strategy used by Murrup-Stewart et al. [26], we conducted an electronic database search to enable a multidisciplinary search outcome (Fig. 1). The databases included PubMed, Informit-Indigenous Studies database, Web of Science, ProQuest, SCOPUS, Ovid Medline, HealthInfoNet, and PsycInfo. The search was informed by the following eligibility criteria:

1. written in English language;
2. articles published between 2003 and 2018 (to ensure currency);
3. studies focusing on Aboriginal or Torres Strait Islander populations;
4. studies that included interventions, programs, projects, or services targeting SEWB among Aboriginal and Torres Strait Islander people; and
5. both qualitative, quantitative, and mixed methods studies. Studies that did not meet the eligibility criteria were excluded from further consideration.

To balance specificity and sensitivity of the search terms, we used a set of key search terms, truncated as appropriate to each database. A combination of the following terms were used and searched for in the title, abstract, and keywords: Aborigin* OR Indigen* OR “First Nations” “Torres Strait”; AND youth OR “young people”; AND Evaluat* OR program OR project OR intervention OR service; AND wellbeing OR well-being OR social OR emotion* OR SEWB OR psychological OR trauma OR stress* OR suicide OR grief OR loss OR healing OR identity OR “mental health” OR “severe and complex mental health needs” OR cultur* OR empowerment OR racis* OR shame* OR discriminat* OR stigma OR disillusion* [26].

We were conscious that the inclusion of the ‘severe and complex mental health needs’ key term could potentially limit our literature search though we applied it deliberately to capture programs and services in this specific and under investigated area.

Relevant articles were selected in three stages based on the eligibility criteria described above. Articles that did not mention mental health, SEWB, or Aboriginal and Torres Strait Islander people in the titles were not included for further consideration. The next step involved removing articles deemed to be irrelevant based on abstract content. This included abstracts that did not explicitly discuss interventions, programs, projects, or services targeting SEWB among Aboriginal and Torres Strait Islander people.

In addition, Google Scholar and websites of relevant organisations were searched to locate grey literature, such as reports and conference papers. The organisational websites included Indigenous Health*Info*Net, Closing the Gap Clearinghouse, Beyond Blue, and The Lowitja Institute. The overall search strategy retrieved 732 potentially relevant articles, which comprised 692 journal articles and 40 grey literature publications. Seventy-one articles were deemed relevant by titles and abstracts. Full texts articles were then obtained for further review. Ten additional relevant articles were identified following the manual scanning of reference lists of these articles. Of the 81 articles identified, 26 met the full eligibility criteria discussed above. The results of the search strategy are illustrated in Fig. 1 below.

This review process involved multiple stakeholders. In alignment with contemporary scholarship about Indigenous data sovereignty and SEWB principles, it was important that Aboriginal and Torres Strait Islander people were represented on the review team. Both DS and ES identify as Aboriginal. Importantly DS was involved in the analytic process underpinning the review, while ES supported the study with critical revision and review, providing an opportunity for two-way learning and cultural capacity building among review team (including HG and NTK).

This was guided by a framework analysis approach. Framework analysis involves coding information against pre-selected themes and is often perceived as a pragmatic approach to real-world investigations [27–29]. It has previously been used successfully in health and education policy contexts [18, 29]. In this instance, we used the SEWB Framework as a benchmark [7], we assessed the extent to which studies identified in the review aligned to the principles of the SEWB Framework [7, 14]. The intention was not to assess the effectiveness of the programs and services at an individual level – as independent evaluations are best to achieve this outcome. We are mindful, however, that there are limitations associated with critiquing older programs and services between 2003 and 2004 against principles outlined in the previous (2004–2009) and current SEWB Framework (2017–2023). Nevertheless, the SEWB Framework was chosen because (1) it is a nationally recognised framework to guide and inform policy development, research, and evaluation into Aboriginal and Torres Strait Islander mental health and wellbeing reforms, (2) it was developed under the auspice of the Aboriginal and Torres Strait Islander Mental Health and Suicide Prevention Advisory Group, and (3) there was strong alignment between principles in the current SEWB Framework with findings and outcomes of those included in previous reports and frameworks (as already outlined above) [1, 2, 5–10, 14].

## Results

Having examined the promising practices associated with SEWB programs and services for Aboriginal and Torres Strait Islander people, we are now reviewing the alignment to the nine guiding principles to discuss the extent to which these programs and services align with the SEWB Framework. However, our main focus was to identify programs targeting Aboriginal and Torres Strait Islander young people with severe and complex mental health needs, we identified a significant gap in the literature in relation to culturally sensitive programs and services targeting this special population. Thus, we have chosen a wider perspective and extended the key search terms to address social and emotional wellbeing and mental health broadly.

### Element 1 (health as holistic)

*Aboriginal and Torres Strait Islander health is viewed in a holistic context that encompasses mental health and physical, cultural and spiritual health. Land is central to wellbeing. Crucially, it must be understood that while the harmony of these interrelations is disrupted, Aboriginal and Torres Strait Islander ill health will persist.*

Many studies valued the influence of holistic interventions on SEWB among Aboriginal and Torres Strait Islander people. They strongly advocated for SEWB programs that were informed by Aboriginal perspectives, rather than Westernised biomedical models [9, 30–43]. For the programs to be effective, studies also highlighted the need for Aboriginal ownership and leadership to be reflected throughout the design, implementation, and evaluation phases; and for the use of culturally-sensitive screening and assessment instruments [44, 45]*.*

Aboriginal cultures are often collectivistic. Therefore, the mental, physical, emotional, and spiritual health and wellbeing of the whole community is vital for the health and wellbeing of the individuals that comprise it [9, 30, 33, 34, 36–42, 46]. Drawing on this, an all-inclusive and multidisciplinary approach based on the restoration of harmony and balance, rather than the reduction of symptoms or restoring of function, was identified as a significant factor in developing resilience, life skills, and improved SEWB [16, 34, 47, 48]. Studies indicated that programs should address social, emotional, physical, spiritual, and cultural needs through a focus on familial and community interconnectedness as well as connections to the environment and the spiritual realm [8, 49, 50]. This was aptly summarised in a qualitative study conducted with Aboriginal young people [47],

“ … unless you were absolutely aware of Aboriginal culture, Aboriginal health, the whole history, socioeconomic conditions and so on, and if you just approached this strictly from a mental health or emotional social wellbeing [viewpoint] without taking all the other issues into account, you could make the wrong decision and therefore subject not only the child, but the parents and everybody, to needless hours of the wrong way of treating it.” ([51]:604)

In a SEWB program delivered in Queensland, the holistic concept of health was incorporated into the program design through focus areas relating to ‘being healthy’; ‘being loved and safe’; ‘personal growth’ and ‘cultural and spiritual healing’. Program components therefore modelled holistic health, with suicide prevention training sessions for young people including activities related to physical activity and on healthy diet and nutrition education [52].

### Element 2 (the right to self-determination)

*Self-determination is central to the provision of Aboriginal and Torres Strait Islander health services.*

Other scholars have argued that successful suicide prevention activities and social-emotional wellbeing programs should also have a focus on community functioning, collective self-determination and cultural continuity [4, 53]. Yet, the principle about the right to self-determination was only partially embedded in a practical sense into programs identified through the review process. Instead, this principle was more commonly discussed in theoretical studies focusing on frameworks [4, 53].

### Element 3 (the need for cultural understanding)

*Culturally valid understandings must shape the provision of services and must guide assessment, care and management of Aboriginal and Torres Strait Islander peoples’ health problems generally and mental health problems in particular.*

Threaded throughout the commentary was the significance of using culturally-appropriate terminology to describe mental illness [38, 39, 51, 54]. A general lack of awareness of the symptoms of mental illness in the community, and the use of technical language by mainstream health services, and the way these services engage with Aboriginal and Torres Strait Islander people to promote mental health literacy, are perceived as consistent barriers to recognise mental illness and health service uptake,

“I don’t think a lot of people know, I think it’s got to be more talked about within the Community. There’s a lot of talk about drugs, a lot of Ice and stuff like that, but you don't see much about say...bi-polar or … depression or schizophrenia” ([54]:410).

We conclude that the need for ‘cultural understanding’ surfaced across all of the reviewed articles, but it was discussed in different ways, including the application of culturally-sensitive screening and assessment instruments [44, 45]; and the employment of Aboriginal trainers and staff [45].

### Element 4 (the impact of history in trauma and loss)

*It must be recognised that the experiences of trauma and loss, present since European invasion, are a direct outcome of the disruption to cultural wellbeing. Trauma and loss of this magnitude continue to have intergenerational effects.*

The experience of trauma and loss and its direct outcomes to wellbeing have been long recognised, and well documented, in Aboriginal and Torres Strait Islander health scholarship [10]. Enduring forms of trauma (historical, cross generational and intergenerational) have harmful effects on the SEWB of Aboriginal people and can lead to, and exacerbate, mental illness [31]. Effective work with Aboriginal clients, therefore needs to acknowledge trauma, but within a culturally safe environment, and in a culturally competent and trauma informed manner [4].

### Element 5 (recognition of human rights)

*The human rights of Aboriginal and Torres Strait Islander peoples must be recognised and respected. Failure to respect these human rights constitutes continuous disruption to mental health (as against mental ill health). Human rights relevant to mental illness must be specifically addressed.*

Recognition of human rights as a basic guiding principle in SEWB services and programs was only partially evident throughout the review. Rees et al’s (2004) article relating to family violence was particularly illuminating. They repositioned family violence among Aboriginal people as a human rights issue. They argued that the family wellbeing empowerment program - implemented in Alice Springs between 1996 and 2002 - could be enhanced by the explicit inclusion of human rights norms and standards into the program as a mechanism to further support participants to challenge health inequality, including violence [55].

### Element 6 (the impact of racism and stigma)

*Racism, stigma, environmental adversity and social disadvantage constitute ongoing stressors and have negative impacts on Aboriginal and Torres Strait Islander peoples’ mental health and wellbeing.*

Accessing mental health services is a stigmatising practice for many Aboriginal people. The intersection between racism and stigma is also perceived as one of the main barriers in discussing problems with family and/or the community [54]. Barriers to discussing problems with family and/or the community included the fear of being ostracised from the community [54] and stigma attached to mental illness [9, 38, 44, 56]. More specifically, Aboriginal men believed that contacting psychiatric services was a ‘shame job’ [10]. Participants in Isaacs et al’s study [54] mentioned the fear of being labelled ‘mental’ by one’s own community if they consulted mental health services,

“As soon as people say, ‘Oh you’ve got a mental health issue!’ there’s an automatic barrier come up... Hey this guy is a little bit freaky and they kind of put you in that little category of black sheep over there” ([54]:410).

Central to not engaging with health services was past negative experiences such as shame and discrimination. The commentary ranged from feeling stereotyped by non-Aboriginal staff, through to anxiety, stress, and fear experienced when accessing mental health services [38–42, 54, 56]. In contrast, the inclusion of Aboriginal staff in health services and mental health programs emerged as a powerful predictor of health service use by Aboriginal and Torres Strait Islander people. This is summarised in Baba et al’s study [57],

“I’ve come to this horrible, negative space [the hospital], but coming here [to the program] is helping me get back … it’s like, for me coming to this program is like I’m on a journey, a journey of healing for me, yeah, to get healthy, stronger and live longer” ([57]:7).

The above-mentioned experience often led to reluctance among Aboriginal people to use mental health services. They also resulted in feelings of mistrust and discomfort; a lack of meaningful communication and engagement with health service providers; non-disclosure of information to staff; misdiagnosis or inappropriate treatment; and non-adherence with treatments/program [9]. To build trust and respectful relationships with clients, and thus improving access to services, studies highlighted the need for staff cultural education of Aboriginal history; placements within Aboriginal organisations; and mentoring with Aboriginal Elders or staff [16, 34, 57, 58],

“I was going to a doctor in Cleveland, and I didn’t feel comfortable there, but being here, where there’s other Aboriginal people around, yeah I felt so comfortable when I came here the first time … there were Aboriginal nurses as well … and you could relate to them a bit more. As if you’re talking to your own daughters or sisters” ([57]:62).

However, it was argued that for some cultural training for non-Aboriginal and Torres Strait Islander people would not always work: *“If you are a highly trained professional who has done cultural competency training that doesn’t mean you are an expert”* ([16]:57).

### Element 7 (recognition of the centrality of kinship)

*The centrality of Aboriginal and Torres Strait Islander family and kinship must be recognised as well as the broader concepts of family and the bonds of reciprocal affection, responsibility and sharing.*

Aboriginal and Torres Strait Islander people’s SEWB is determined by a range of inter-related domains such as: body, mind and emotions, family and kinship, community, culture, country, and spirituality [53]. Among the articles, commentary about kinship and extended Aboriginal family systems were reflected in articles relating to a SEWB Framework [53] and in a qualitative study [51]. The latter clearly emphasised the importance of the extended family and kinship to support mental health and wellbeing of Aboriginal young people.

Another theme that emerged across multiple studies was the concept of yarning, which is a valued decolonisation research method giving space to Aboriginal participants to communicate from their frames of reference and also centres their knowledge, values, and perspectives [59–61]. Participants found yarning circles as an appropriate and safe way to tell and share stories and information. [8, 9, 37, 38, 42, 46]. Sharing the vicissitudes of life, through various activities, resulted in a feeling of belonging and encouraged people to express their emotions without shame or getting ‘labelled’. Activities such as storytelling, painting, art, and music, which are central to Aboriginal culture, also helped people to develop coping mechanisms learned from others in the circle, and thus facilitated social and emotional wellbeing [44, 62]:

“We wouldn’t tell anybody about our secrets, what is happening in community, but lately when [we] have been doing the workshops, everyone is telling stories openly; bringing it out and telling sad story, lonely story and Tjukurpa [Law]. It makes us comfortable, happy. We feel it that way” ([44]:724).

However, this approach did not appear to work in situations where Aboriginal and Torres Strait Islander people were talking to non-Aboriginal people. It seemed non-Aboriginal people had difficulty in relating to the language and cultural differences of their Aboriginal and Torres Strait Islander counterparts. As Poroch notes “it is harder for Aboriginal people to be able to talk to white people as they do not relate to Aboriginal people” ([63]:42). In these instances, Aboriginal and Torres Strait Islander people were required to develop coping behaviours, which ultimately contributed to a reduction in SEWB [54, 63].

### Element 8 (recognition of cultural diversity)

*There is no single Aboriginal or Torres Strait Islander culture or group, but numerous groupings, languages, kinships and tribes, as well as ways of living. Furthermore, Aboriginal and Torres Strait Islander peoples may currently live in urban, rural or remote settings, in urbanised, traditional or other lifestyles, and frequently move between these ways of living.*

Description about cultural diversity really only surfaced in studies discussing SEWB frameworks [5, 53]. This was articulated through statements implying that wellbeing is supported by locally defined, culturally safe primary healthcare services, that create culturally safe and welcoming places, develop a trusting relationship with clients and communities, acknowledge, understand and accept cultural diversity, and deliver flexible primary health care services [5]. The need to acknowledge the diversity between and within Aboriginal and Torres Strait Islander communities was also mentioned, but according to participating Aboriginal healthcare professionals, was often overlooked [5]. Cultural diversity was also articulated through some domains of SEWB such as connection to community and culture. It was highlighted that despite the diversity and fluidity; Aboriginal people are strongly connected with a community and strengths of the community and cultural domains of SEWB are reflected by factors such as cultural diversity [53].

While multiple culturally adapted screening tools are available [64], there is a concern that screening should occur as just one part of a broader culturally competent assessment process [65]. In another study, low screening rates suggest that SEWB concerns may be under-diagnosed, and that the gateway to SEWB service provision is therefore limited [66].

In some articles a strengths-based approach was clearly articulated and applied through program delivery [16, 67, 68]. For instance, Gibson’s (2018) approach included the following key dimensions, listen respectfully to the person; build genuine relationships; use appropriate communication skills; critically reflect on Australia’s political, historical and social context; apply a human-rights based approach; and finally, evaluate the processes and outcomes [67].

The inclusion of capacity building in programs directed at improving SEWB constituted powerful influences on SEWB [9, 33, 36–42]. The results from the Men’s Groups and Sheds study suggested that Aboriginal men were enthusiastic about participating in formal education, and career and personal development or training [45]. However, participants indicated that training programs needed to dovetail well with their culture and traditions or be cross-cultural in focus [45]. There was also an emphasis on the trainers being Aboriginal or those who understand Aboriginal cultures, as voiced by participants in the Men’s Groups and Sheds study, *“We want Aboriginal men to be educated as counsellors...we need a culturally specific program”* and *“we’ve got to be smart...if we are better educated we can do better for our families”* ([45]:612). This suggests there is a compelling evidence base of the need for a strong Aboriginal and Torres Strait Islander workforce.

### Element 9 (recognition of Aboriginal strengths)

*It must be recognised that Aboriginal and Torres Strait Islander peoples have great strengths, creativity and endurance and a deep understanding of the relationships between human beings and their environment.*

Enhanced confidence, self-esteem, and a sense of belonging were discussed positively as outcomes from participation in culturally appropriate SEWB programs and services addressing Aboriginal strengths. For example, a study about the *Ngala Nanga Mai* parent group program discovered that all program activities were strengths-based. The positive and safe space that was created for the participating parents allowed them to discover their new creative strengths in art, education, and parenting that increased their satisfaction and confidence [69]. In addition, program participants described themselves becoming more empowered in many aspects of their life such as achieving higher inner strengths, more powerful sense of community, holistic acknowledgment of their children’s health, and capacity to prevent illnesses [69].

Building on strengths were found to be central to the development of resilience; had a positive effect on identity and motivation; assisted in recognising the causes of problems, including an enhanced sense of self-control; and thus, resulted in improved SEWB. As Jersky et al. reported,

“It’s helping me, giving me encouragement...and willpower to actually get out and do something...I have started to think about my life and what's ahead of me” ([69]:118).

Building relationships with the services, and the need for integrated services, such as Centrelink, Medicare, and Housing, and providing services focused on addressing the social determinants of health, was valued in improving people’s health in a holistic sense. This was also recognised as a conduit for addressing the gap between Aboriginal and non-Aboriginal people’s social, economic, cultural, and political development and aspirations. This, in turn, was found to be conducive to maximising health and wellbeing outcomes, particularly among Aboriginal and Torres Strait Islander men, highlighting the importance of gender-sensitive approaches in this realm [45, 70, 71]. This was reflected in below participant quote from an Indigenous Men’s Groups and Sheds study,

“...we need men in trouble to be referred to men’s groups...don’t send them to prison...we want to run programs for them in the Sheds’ but to do that...you guys need a relationship with Magistrates” ([45]:611).

Murphy et al’s (2004) appreciative inquiry of the Aboriginal Youth Arts and Culture Project (IYACP) demonstrated a significant positive and holistic impact on the SEWB Aboriginal and Torres Strait Islander individuals, service providers and the broader community through allowing participants to identify personal and community strengths, assets and aspirations. It fulfilled its aim to support and develop pride, self-esteem, skills, creativity and leadership in the local Aboriginal community [71].

## Discussion

In the recent scoping review our intention was to describe how parts of the identified key practices that aim to improve SEWB among Aboriginal people, including a special focus on young people with severe and complex mental health issues attempted to address the principles from the SEWB Framework. The aim was to emphasise the considerable value of the implementation of these guiding principles and highlight their potential contribution to service design.

Most of the identified programs included Aboriginal and Torres Strait Islander perspectives that address restoring harmony, balance and holistic context of health (Principle 1) [30, 44, 45, 51, 52]. For instance, the United Health Education and Learning Program (UHELP) [52] and The Uti Kulintjaku Project (UK Project) [44]. In addition, locally defined, culturally safe health care practices and assessment tools (Principle 8) [16, 56, 64, 66, 67] such as *Here and Now Aboriginal Assessment* (HANAA) [64] and *The Cultural Yarn* tool [16], as well as programs focusing on community functioning (Principle 9) [34, 53, 69–71] like *Indigenous Youth Arts and Culture Project* (IYACP) [71], *National Empowerment Project* (NEP) [53], and *Ngala Nanga Mai* (We Dream) [69] were widely discussed. Whereas, recognition of family violence as a response to and reflection of historical and intergenerational trauma (Principle 4) [4, 10, 31, 45], and the need of building trustful and respectful relationships with clients and understanding the enablers and barriers of programs in particular settings (Principle 2) [16, 31, 54, 58] were less addressed explicitly by the identified initiatives. However, *Strong Women, Strong Babies, Strong Culture Program* [31], *Aboriginal and Torres Strait Islander Suicide Prevention Project* (ATSISPEP) [4], *Men’s Groups and Sheds* [45], and *The Family Wellbeing Empowerment program* [58] were found to be great exceptions. While, program alignment with the acknowledgment of interconnectedness within family and community (Principle7) [53, 72], engaging Aboriginal Torres Strait Islander health practitioners, traditional healers and healing experts as health consultants (Principle 3) [53, 57], and involving capacity building for effective connections to and relationships with social services (Principle 5) [55, 67] were less visible in the identified programs, *Family Wellbeing Program* [55] and *National Empowerment Project* (NEP) [53] incorporated these principles.

Even though evidence showed that racism has a destructive impact on behaviours, life outcomes [73] as well as physical and mental health [74], among the guiding principles, recognising cultural notions of mental health including social and emotional wellbeing and understanding and supporting Aboriginal and Torres Strait Islander people within communities, as well as broader community, understanding, and knowledge to prevent racism, stigma, and shame (Principle 6) [10, 54, 57], was found to be the least addressed principle in the identified programs. Only *Koori Kids Mental Health Network* [10] and *Young People’s Mental Health* [10] initiatives were identified in the reviewed literature as good examples for covering this principle. Previous literature underpinned that as a result of the complexity of the racism phenomenon, besides group context, inter-personal and systematic racism also needs to be addressed and discussed in programs [73–76].

Among the identified literature, most of the initiatives incorporated some principles at the same time, *YouthLink* was a good example of embedding all the nine ones explicitly and simultaneously [34] (Table 2).

**Table 2:** The nine guiding principles with examples and their implication for practice *(National Strategic Framework for Aboriginal and Torres Strait Islander Peoples’ Mental Health and Social and Emotional Well Being 2017-2023)* [7, 9, 10, 14]

Guiding principle	Description	Implications for practice	Selected examples within the literature	Selected literature references and initiatives (e.g. program/service/activities, practices and research)
**1. Health as holistic**	Aboriginal and Torres Strait Islander health is viewed in a holistic context that encompasses mental health and physical, cultural and spiritual health. Land is central to wellbeing. Crucially, it must be understood that while the harmony of these interrelations is disrupted, Aboriginal and Torres Strait Islander ill health will persist	Health and well-being address Aboriginal and Torres Strait Islander ways of knowing and being; recognition that identity is central to health outcomes.	Programs are multidisciplinary; embed Aboriginal and Torres Strait Islander perspectives that restore harmony and balance rather than western biomedical models.	Garvey 2008 (e.g., *SEWB preventive services such as MindMatters, The Family Wellbeing)* Williamson et al. 2010 *(a qualitative study at ACCHOs in Sydney about Aboriginal child* *and adolescent mental health)* Southcombe et al. 2015 *(Men’s Groups and Sheds)* Togni 2017 *(The Uti Kulintjaku Project) (UK Project)* Skerrett et al. 2018 *(United Health Education and Learning Program) (UHELP)*
**2. The right to self-determination**	Self-determination is central to the provision of Aboriginal and Torres Strait Islander health services.	Aboriginal and Torres Strait Islander ownership of program design, implementation and evaluation; ACCHOs provide culturally safe places for service delivery.	Understand how and why a program works within a particular community or setting; Build trusting and respectful relationships with clients; include lived experience councillors.	Bamblett et al. 2012 *(*e.g.*, The Cultural Yarn tool)* Day &Francisco 2013 *(*e.g.*, Strong Women, Strong Babies, Strong* *Culture Program)* Isaacs et al. 2013 (*Qualitative Description as a study design and qualitative study about mental health clients)* Whiteside et al. 2014 *(The Family Wellbeing Empowerment program)*
**3. The need for cultural understanding**	Culturally valid understandings must shape the provision of services and must guide assessment, care and management of Aboriginal and Torres Strait Islander peoples’ health problems generally and mental health problems in particular	Framework and practice guideline founded on Aboriginal and Torres Strait Islander notions of SEWB including body, mind and emotions, family and kinship, community, culture, country and spirituality; Workforce training that includes Aboriginal and Torres Strait Islander capacity building as well as culturally informed training for the broader workforce.	Reduce misdiagnosis due to overcrowding, hunger, grief and learning difficulties and other social issues must be addressed; Engage and development of Aboriginal and Torres Strait Islander health practitioners, traditional healers and healing experts as health consultants.	Baba et al. 2014 *(a qualitative study with clients of AMS and ACCHS in Brisbane about self-identified health needs)* Dudgeon et al. 2017 *(qualitative data analysis from the National Empowerment Project) (NEP)*
**4. The impact of history in trauma and loss**	It must be recognised that the experiences of trauma and loss, present since European invasion, are a direct outcome of the disruption to cultural wellbeing. Trauma and loss of this magnitude continue to have intergenerational effects.	Trauma informed practice and professional learning within the workforce.	Recognise family violence as a response to and reflection of historical and intergenerational trauma; Impact of high levels of incarceration and interactions with the criminal justice system.	Day and Francisco 2013 *(*e.g.*, Strong Women, Strong Babies, Strong Culture Program)* Dudgeon and Holland 2018 *(Aboriginal and Torres Strait Islander Suicide Prevention Project) (ATSISPEP)* Southcombe et al. 2015 *(Men’s Groups and Sheds)* Swan & Raphael, 1995 *(*e.g.*, Narrative Therapy, Family Therapy)*
**5. Recognition of human rights**	The human rights of Aboriginal and Torres Strait Islander peoples must be recognised and respected. Failure to respect these human rights constitutes continuous disruption to mental health (as against mental ill health). Human rights relevant to mental illness must be specifically addressed.	Community and individual empowerment and capacity building.	Understand the impact of family violence through family focused programs; Include capacity building for effective connections to and relationships with social services.	Rees et al. 2004 *(evaluation of an Indigenous empowerment program (Family Wellbeing Program), human rights framework)* *Gibson 2018 (yarning about culturally responsive services, development of Indigenous Research Paradigm)*
**6. The impact of racism and stigma**	Racism, stigma, environmental adversity and social disadvantage constitute ongoing stressors and have negative impacts on Aboriginal and Torres Strait Islander peoples’ mental health and wellbeing	Understanding and supporting Aboriginal and Torres Strait Islander within communities, as well as broader community, understanding and knowledge to prevent stigma and shame	Recognise cultural notions of mental health including social and emotional wellbeing	Isaac et al. 2013 (*Qualitative Description as a study design and qualitative study about mental health clients)* Swan & Raphael 1995 (*Koori Kids Mental Health Network, Young People’s Mental Health)* Baba et al. 2014 *(a qualitative study with clients of AMS and ACCHS in Brisbane about self-identified health needs)*
**7. Recognition of the centrality of kinship**	The centrality of Aboriginal and Torres Strait Islander family and kinship must be recognised as well as the broader concepts of family and the bonds of reciprocal affection, responsibility and sharing.	Social and emotional wellbeing established within a family and community focus including assessment and programs that encompasses family kinship networks.	Acknowledgment of interconnectedness within family and community; including environmental and spiritual connections.	Dudgeon et al. 2017 *(qualitative data analysis from the National Empowerment Project) (NEP)* Williamson et al. 2010*(a qualitative study at ACCHOs in Sydney about Aboriginal child* *and adolescent mental health*
**8. Recognition of cultural diversity**	There is no single Aboriginal or Torres Strait Islander culture or group, but numerous groupings, languages, kinships and tribes, as well as ways of living. Furthermore, Aboriginal and Torres Strait Islander peoples may currently live in urban, rural or remote settings, in urbanised, traditional or other lifestyles, and frequently move between these ways of living	Culturally adaptive screening and assessment	Develop locally defined, culturally safe primary health care.	Davey et al. 2017 *(Framework development)* Langham et al. 2017 *(cross-sectional analysis of Indigenous records)* Janca et al. 2015 (Here and Now Aboriginal Assessment (HANAA) Bamblett et al. 2012 *(*e.g.*The Cultural Yarn tool)* Eley et al. 2007 *(survey about mental health service needs)* Gibson 2018 *(yarning about culturally responsive services, development of Indigenous Research Paradigm)*
**9. Recognition of Aboriginal strengths**	It must be recognised that Aboriginal and Torres Strait Islander peoples have great strengths, creativity and endurance and a deep understanding of the relationships between human beings and their environment	Promotion of resilience and self-control through culturally informed practices; Support and develop pride, self-esteem, skills, creativity	Design Men’s and Women’s health, Youth Arts and Culture projects; Capacity building that produces supportive networks; Programs focus on community functioning, collective self-determination and cultural continuity.	Jersky 2016 *(Ngala Nanga Mai;* *We Dream)* Murphy et al. 2004 *(Indigenous Youth Arts and Culture Project) (IYACP)* Dudgeon et al. 2017*(qualitative data analysis from the National Empowerment Project) (NEP)* Tsey et al. 2007 *(*e.g. *Family Wellbeing project, Cape York Substance Misuse Strategy, Indigenous Men’s Support Groups)* Sabbioni et al. 2018 *(YouthLink)*

The review also identified three other guiding principles that are not explicitly expressed in the current national strategic framework. These include ‘a commitment to capacity building’ [45]; ‘individual skill development’ [69, 71]; and the ‘risk of development of maladaptive coping mechanisms’ [54, 63]. In reality, there were only a few articles focused on training programs and services specifically targeting Aboriginal young people.

None of the reviewed initiatives had a specific focus on Aboriginal and Torres Strait Islander young people with severe and complex mental health needs. Usually they applied a broader approach targeting social and emotional wellbeing. Although, the results showed that the young people presented at *YouthLink* had severe clinical presentations involving multiple psychiatric diagnoses, previous hospital admissions, high rates of childhood trauma, self-harm, substance abuse, and homelessness. This represents a significant gap in the evidence-base about how best to support the SEWB needs of this particularly vulnerable group. Future studies and programs should pay greater to addressing this significant gap.

It was also identified that in program and service evaluation processes evidence-based approaches were preferred. Such approaches may be conceptually dissonant with Aboriginal knowledges and practices, which can raise concerns about the cultural and social appropriateness of mainstream outcome measures [10, 34, 77, 78]. A recent review found that less than 10% of Aboriginal oriented programs are evaluated [79]. Where evaluations were conducted many lacked a suitable approach and measurement [80]. Indeed, the Productivity Commission has recently been tasked to develop an Aboriginal Evaluation Strategy to consider how Australian Government agencies can work better with Aboriginal and Torres Strait Islander organisations to deliver improved evaluation outcomes and to use evaluation findings and recommendations more effectively [81]. It will be achieved by:

- Developing a principles-based evaluation framework for policies and programs;
- Analysing and identifying Aboriginal evaluation priorities and principles; and
- Identifying the processes and institutional aspects needed to assist the adoption and success of the Indigenous Evaluation Strategy

The lack of the culturally appropriate outcome measures was identified as a limitation in the review findings. Hence, further efforts are required to improve Aboriginal evaluation processes, particularly among mental health services where standardised measures are mandatory [34].

The present scoping review has some limitations in its process. For instance, (1) it was carried out as a team work and diverse views created challenges in the presentation and interpretation of data; (2) the research team included two Aboriginal and Torres Strait Islander researchers, reflecting the need of increasing Aboriginal ownership regarding SEWB; and (3) review findings have not been shared with a wider network of Aboriginal and Torres Strait Islander SEWB workforce for review, and (4) the review against the principles is subjective, to the extent that it is impacted by the individual understandings and concepts of racism, self-determination, and trauma and loss, within the review team. However, Aboriginal and Torres Strait Islander were directly involved in the analysis, and key Aboriginal and Torres Strait Islander scholarship and policy documents have been used to ground this review.

### Implications for practice

The services, and programs that were deemed to be most effective, were those that recognised a persons’ right to self-determination and those which were culturally responsive. They focused not just on understanding these rights and needs but also point out the negative effects originating from intergenerational trauma, grief and loss. Focusing on the positive effects of kinship and communities, and adopting a strengths-based approach, were also deemed to be important.

The scoping literature review also indicated that the following elements were critical considerations for improved service delivery in Aboriginal mental health and wellbeing contexts:

- involvement of families and caregivers with patient/client consent [35];
- an acknowledgement of the role of the community in mental health promotion [35];
- greater interprofessional collaboration and information sharing [35];
- improved access to specialist services [32, 34];
- involvement of Aboriginal Practitioners (AP) who can provide direct case management, clinical treatment and responsive presence within family and community networks [34];
- integrated team care packages tailored to individual needs [32];
- improved mental health system implemented in partnership with consumers, carers, mental health stakeholders and state and territory governments [32];
- culturally safe environment [33];
- application of an integrated educational/healing model (educaring approach) [33];
- providing support for professionals and for people to build their community [33]; and
- providing trauma healing program in early childhood [33].

The National Empowerment Program (NEP) which was conducted at nine sites - Cherbourg (Queensland); Kuranda (Queensland); Darwin (Northern Territory); Sydney (New South Wales); Toomelah (New South Wales); Mildura (Victoria); Perth (Western Australia); (Northam/Toodyay (Western Australia) and Narrogin (Western Australia) - summarised the key issues and recommendations that were compiled through the community consultation and social-emotional workshops in this way:

1. Principles (program needs to be community owned and culturally appropriate);
2. Delivery (any program should be flexible and delivered on country, where possible);
3. Content (the content of programs should include modules that address cultural, social and emotional wellbeing, healing, and self-empowerment).

It was also found that in Aboriginal healing programs [8] regardless of where they were located, who they were servicing, or what outcomes they were working towards achieving, the following eight critical elements were advocated:

1. proactive rather than reactive;
2. incorporate strong evaluation frameworks;
3. developed to address issues in the local community;
4. driven by local leadership;
5. have a well-developed evidence-base and theory base;
6. combine Western methodologies with Aboriginal healing;
7. understand the impact of colonisation and transgenerational trauma and grief;
8. build individual, family and community capacity [8].

As such, it seems that the effectiveness of current SEWB programs and services targeting Aboriginal and Torres Strait Islander young people is challenged and limited by the following six needs. To improve the effectiveness of the SEWB programs among Aboriginal people, these needs have to be addressed:

#### Strengths-based approaches that promote cultural identity

The need for greater recognition of the extreme circumstances that these young people are growing up within, and for the value of strengths-based holistic approaches that promotes cultural identity as a necessary component of successful ways forward.

#### Growth in the Aboriginal mental health workforce

The need to seriously address Aboriginal workforce shortages, especially the employment of Aboriginal people with relevant lived experiences and skills to fill the critical roles required.

#### A long-term outlook

Avoid reactive approaches that respond to immediate stress and crisis and invest in proactive health-promoting approaches that build young people’s resilience and address the social and cultural determinants of health and wellbeing, supporting a long-term vision.

This will require longer-term commissioning cycles for many young people -oriented, and Aboriginal and Torres Strait Islander, mental health services.

#### Enhanced co-ordination and communication between services

There is a need to reduce systematic barriers, and promote practical pathways, to help to link organisations to work collaboratively and effectively around the young people’s support needs. This includes building relationships and communication across sectors.

#### Stronger monitoring and evaluation

The systematic monitoring and evaluation of the processes, impacts, and outcomes of SEWB programs and services is important. These need to be respectful of young people’s voices and aligned with Aboriginal methodologies.

#### More targeted policies and practice guidelines

The need for more targeted policies and practice guidelines to improve decision-making, funding distribution, resources, and commitment to help plan and implement quality SEWB programs suitably tailored to Aboriginal and Torres Strait Islander young people is urgently needed [15, 82].

The abovementioned needs cut across government, non-government and local community organisations that seek to support programs and services aiming to promote SEWB among Aboriginal and Torres Strait Islander young people in an effective and sustainable manner. Efforts to address these six areas could make a significant difference to improving SEWB outcomes over the longer-term.

## Conclusion

There is clear evidence about the disproportionate burden of mental health concerns experienced among Aboriginal young people [32, 83]. Yet, evidence about the effectiveness of current SEWB programs and services targeting Aboriginal and Torres Strait Islander young people is still scant though there are some excellent studies. For instance, Palmer’s three-year evaluation study about the well-respected, award-winning, Yiriman Project that offers different types of ‘on-country’ trips where young people can follow in the footsteps of Elders [84]. Furthermore, Tighe and McKay’s (2012) study about a Kimberly suicide prevention program called ‘*Alive and Kicking Goals!’* that supported Aboriginal youth through peer education and leadership training [85]. Haswell et al’s report about SEWB of Indigenous youth that identified and highlighted critical success factors such as effectiveness, sustainability, growth, and societal factors in successful service delivery is also crucial [48] as well as reviews about Warlpiri Youth Development Aboriginal Corporation (WYDAC) youth programs [86]. As such, very little is known about the ideal pathways for the treatment of Aboriginal and Torres Strait Islander young people with severe and complex mental health needs [35, 48]. We suggest for commissioners of programs and services and practitioners to focus on the need for commitment of the nine guiding principles and guidelines with a clearer understanding of the SEWB model and the interrelationship of the seven interrelated domains before they fund services since they are critical to individual and community wellbeing. We argue the selective application of nationally agreed principles articulated in SEWB frameworks, alongside a paucity of scholarship relating to Aboriginal young people-specific SEWB programs and services, is impinging on the development of promising practice in this space. We suggest the inclusion of adequate resources for a specific application of all nine principles and to address the above-mentioned needs. Innovative and culturally-informed approaches to the design of SEWB services and programs, complemented by participatory action research and developmental evaluation approaches, will help to build the necessary evidence-base to improve SEWB health outcomes among Aboriginal and Torres Strait Islander young people in Australia. Given the paucity of scholarship into the promising practices aimed at addressing social and emotional wellbeing of Aboriginal and Torres Strait Islander young people, robust research and evaluation approaches are required to generate the relevant evidence-base and inform the development of a nationally recognised promising practice guide. For instance, future work should document the commissioned SEWB programs and services being delivered to Aboriginal and Torres Strait Islander young people to identify the most successful elements and operational principles of identified programs.

## Data Availability

The datasets supporting the conclusions of this article are included within the article.

## Abbreviation

SEWB: Social and Emotional Wellbeing
